# Spatial Genetic Structure and Pathogenic Race Composition at the Field Scale in the Sunflower Downy Mildew Pathogen, *Plasmopara halstedii*

**DOI:** 10.3390/jof8101084

**Published:** 2022-10-14

**Authors:** Abdelhameed Elameen, Denis Tourvieille de Labrouhe, Emmanuelle Bret-Mestries, Francois Delmotte

**Affiliations:** 1 NIBIO, Norwegian Institute of Bioeconomy Research, Division of Biotechnology and Plant Health, N-1431 Ås, Norway; 2 Sainte-Anastasie, Le Bourg, 15170 Neussargues en Pinatelle, France; 3 Terres Inovia, Domaine de Magneraud, 17700 Saint-Pierre-d’Amilly, France; 4 INRAE, Bordeaux Sciences Agro, ISVV, SAVE, 33140 Villenave d’Ornon, France

**Keywords:** downy mildew, *Helianthus annuus*, oomycetes, plant pathogens, race evolution, SNPs, SSRs

## Abstract

Yield losses in sunflower crops caused by *Plasmopara halstedii* can be up to 100%, depending on the cultivar susceptibility, environmental conditions, and virulence of the pathogen population. The aim of this study was to investigate the genetic and phenotypic structure of a sunflower downy mildew agent at the field scale. The genetic diversity of 250 *P. halstedii* isolates collected from one field in southern France was assessed using single-nucleotide polymorphisms (SNPs) and single sequence repeats (SSR). A total of 109 multilocus genotypes (MLG) were identified among the 250 isolates collected in the field. Four genotypes were repeated more than 20 times and spatially spread over the field. Estimates of genetic relationships among *P. halstedii* isolates using principal component analysis and a Bayesian clustering approach demonstrated that the isolates are grouped into two main genetic clusters. A high level of genetic differentiation among clusters was detected (*F*_ST_ = 0.35), indicating overall limited exchange between them, but our results also suggest that recombination between individuals of these groups is not rare. Genetic clusters were highly related to pathotypes, as previously described for this pathogen species. Eight different races were identified (100, 300, 304, 307, 703, 704, 707, and 714), with race 304 being predominant and present at most of the sites. The co-existence of multiple races at the field level is a new finding that could have important implications for the management of sunflower downy mildew. These data provide the first population-wide picture of the genetic structure of *P. halstedii* at a fine spatial scale.

## 1. Introduction

*Plasmopara halstedii* is an oomycete plant pathogen causing downy mildew on sunflowers (*Helianthus annuus*), first detected in Indiana, Iowa, and Minnesota in the early 1920s [1] (Young and Morris 1927). The disease was first described in Europe [2], where it had been introduced with sunflower seeds, in the 1970s. *P. halstedii* is an obligate biotroph oomycete, homothallic and capable of sexual or asexual reproduction [3,4,5,6]. Due to changes in cultural systems and the introduction of new, resistant cultivars, the pathogen has developed many races that have now become a serious problem affecting sunflower fields in Europe. The formation of oospores increases the potential for the long-term survival of the pathogen in the soil [3]. Oospores represent a stable resting phase, independent of the host, and sexual reproduction is likely to increase the adaptability of the organism. The yield losses in sunflower crops caused by *P. halstedii* can be up to 85–100%, depending on the cultivar susceptibility, environmental conditions, and virulence of the pathogen population [7,8,9,10]. Since 1992, *P. halstedii* has been subject to quarantine regulations in the European Union (directive 92/103/CEE), but in 2019 was removed from the list [11]. Since 2006, the number of *P. halstedii* races has increased from 35 [12] to 41 in 2014 [13] and 50 in 2018 [14,15,16,17]; the highest genetic diversity of the pathogen was reported in Canada, the USA, and France [18]. The disease was first detected in France in 1966 [19]. Only one race, 100, was present, and was widely distributed across France. In recent years, downy mildew has become a serious problem for the sunflower crop in France; until 2008, only 16 out of 50 worldwide races had been detected in various regions, suggesting that the pathogen population has evolved rapidly, resulting in a high diversity in virulence [13,14,20,21,22,23]. Furthermore, the wide use of fungicide to control this plant pathogen has led to the development of resistant strains that have decreased the fungicide’s efficacy. Strains of *P. halstedii* tolerant to metalaxyl-M have been described in Hungary [20,24], France [25], the USA [26], Spain [27], and Germany [28]. Combined with suitable agronomic practices, breeding of resistant genotypes is one of the most effective strategies in the management of downy mildew disease in sunflowers [8,10,18,29,30,31,32,33,34,35,36,37,38].

The area of plant pathogens and their evolution, and the consequences on genetic diversity, remain controversial and more studies are required to understand the mechanisms for the development of new races. Our understanding of the recurrent breakdown of major resistance genes in sunflowers can be improved by new findings concerning the key processes governing the evolution of *P. halstedii* populations. The genetic structure of *P. halstedii* has previously been investigated using various molecular approaches, including random amplified polymorphic DNA (RAPD) [39,40,41], inter simple sequence repeats (ISSR) [42], simple sequence repeats (SSR) [43], and internal transcribed spacer (ITS) sequences [4,44]. The focus has been on the use of microsatellites [45,46,47,48,49] and on single-nucleotide polymorphisms (SNPs) through the development of NGS technologies [45,46]. SNPs are more favorable than other DNA dominant markers because dominant marker-based genotyping assays have limited possibilities of multiplexing and low efficiency compared to the automated robustness of the multiplexed SNPs assays. Furthermore, genomic and transcriptomic data of *P. halstedii* have provided new results on genome organization, evolution, and secondary metabolism, and effector proteins [6,32].

*P. halstedii* remains one of the major pathogens on sunflowers and has been showing increasing adaptability, so it is important to monitor its diversity, especially in areas of high sunflower cultivation. Previous studies on *P. halstedii* have revealed considerable diversity [46,50], indicating that hybridization has been going on for many years, at least in the regions that were analyzed in previous studies. *P. halstedii* populations in France have been known to undergo rapid virulence evolution [13,17,46,50,51], but knowledge of the genetic diversity of the pathogen population in a single small field is lacking. To gain better insight into *P. halstedii* and to analyze the consequences of more than two decades of hybridization and rapid virulence evolution, we conducted a large study on the genetic diversity of *P. halstedii*, covering a single field of sunflowers in the main growing regions in France. Although the study was performed in 2008, the results are still important to our understanding of the development of pathogen races. Therefore, the goals of this study were to (i) investigate the spatial genetic structure of 250 *P. halstedii* isolates collected from an infected field in southern France, (ii) address the co-existence of races of *P. halstedii* at the field level, and (iii) combine genetic and phenotypic data to gain insight into the reproductive mode of *P. halstedii* and the evolutionary processes that are leading to the emergence of new virulence profiles.

## 2. Materials and Methods

### 2.1. P. halstedii Isolates Sampling

**Figure 1:**
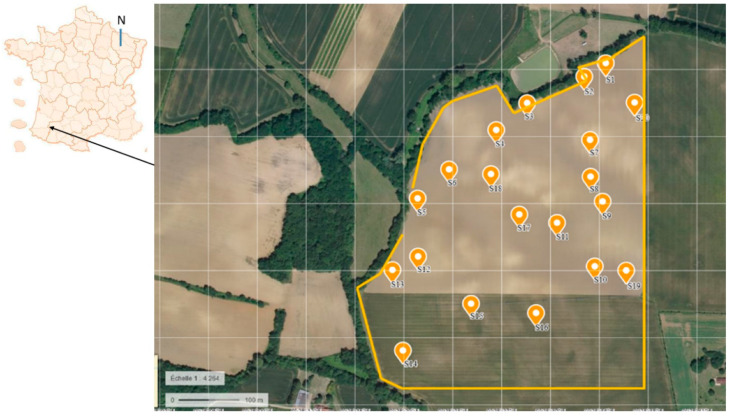
The geographical location of the study in south France and the spatial distribution of the 20 sites (S1 to S20) of *P. halstedii* isolates in the sunflower field (satellite image from https://www.geoportail.gouv.fr/ accessed on 5 September 2022).

A total of 250 isolates of *P. halstedii* (Table S1) were collected from soil samples in May 2008 from a field cultivated with sunflowers where heavy infection of downy mildew was observed in 2006. The area of the field is 15,580 m^2^. The field was located in the most important French sunflower production area, Gers, Saint Puy, a place called «La Poète» in southern France (Figure 1). The field was cultivated according to conventional agriculture practices until 2007 and converted to organic in 2008. The field has been cultivated with sunflowers for at least 10 years of short rotation wheat/sunflower, with high sunflower downy mildew incidence in 2007. With clay–limestone soil, the field presents areas of slow drainage, and several wet spots are present, conditions that are favorable to downy mildew. Since 1999, the sunflower varieties grown in the field were resistant to the following four races: 100, 304, 703, and 710, and, at best, to eight of the nine races officially recognized in France in 2008 (a list that has not changed since).

The soil was sampled on 6 May 2008, before planting the sunflowers, at a depth of 2 to 8 cm (i.e., on the soil horizon where the infections take place), at 20 sites (S), coded S1 to S20. From each site (30 m^2^), six soil samples, 1 L each, were collected. Each soil sample was directly placed in a pot (SOPARCO, 11 cm × 11 cm × 11 cm). To avoid any risk of mixing between samples, each pot was immediately placed in a hermetically sealed plastic bag (Ziploc, 27 × 28 cm). From each liter, 1–5 subsamples were analyzed (except from S16, where only 4 subsamples were analyzed, and from S6, where 31 subsamples were studied). The spatial distribution of soil samples, determined by their exact global position coordinates (GPS), is shown in Figure 1.

### 2.2. Soil Bioassay

The presence of downy mildew inoculum was investigated using the soil test method developed by Tourvieille et al. (2012) [52]. Twenty seeds of the trap genotype, the sunflower line GB without any known resistance gene, were sown in each pot, covered by 1 cm of soil, and grown at 18 °C. After 48 h, which was the time required to obtain 0.5–1.0-cm-long germs, each pot was separately immersed in water for 6 h. Then, the pots were maintained at 18 °C with a 16 h photoperiod (12,000 lux) per day. After 12 days, sporulation was induced by covering the infected seedlings with a transparent plastic bag (PEBD 50 µm) for 48 h to provide saturated humidity [53]. For each of the 120 pots, we registered the number of noninfected and infected seedlings (sporulation) on the cotyledons and/or hypocotyledons. Then, these two measurements, pooled for each site, allow us to estimate the attack rate as the ratio of the total number of emerged seedlings to the infectious potential of each site. The recovery of isolates was performed first, by molecular genotyping (see Section 2.4): depending on the number of diseased seedlings in each pot, a sporulating cotyledon was collected on 1 to 5 seedlings per pot and was lyophilized. Secondly, we performed phenotyping of downy mildew isolates by selecting one cotyledon from 1–6 sporulating plantlets per site. Each pool of cotyledons was used to infect a batch of 4–5 seeds of the GB sunflower line for inoculum multiplication and race identification, as described in Section 2.3.

### 2.3. Race Phenotyping and Nomenclature

Since the phenotyping of races is time-consuming, a subset of 62 *P. halstedii* isolates, representing each of the 20 sites, was characterized for their virulence profiles. *P. halstedii* races were defined on the basis of virulence profiles on a set of nine differential lines of host plants carrying different *Pl* resistance genes (Table 1), as described by Gulya et al. (1998) [54] and Tourvieille de Labrouhe et al. (2000) [55].

Race phenotyping and denomination (Table 1) were performed as described in Cohen and Sackston, 1974 [56], with the modifications proposed in Mouzeyar et al. (1993) [57]. Ten seeds for each of the differentials were sown in each pot, and, 15 days after inoculation, plants were incubated for 48 h in a saturated atmosphere. Plants were deemed susceptible if sporulation was observed in the cotyledons and leaves, and as resistant if no sporulation or only light sporulation was seen on cotyledons. The results of race phenotyping were confirmed by using positive control races (100, 703, and 710) that were previously phenotyped, and by repeating the analyses of 10 randomly selected isolates of *P. halstedii* using the same method. Note that the identification of *P. halstedii* pathotypes today remains based on the nine differential lines used in this work.

### 2.4. DNA Extraction and Polymerase Chain Reaction (PCR)

DNA was extracted from infected plant tissue as previously described for *Plasmopara viticola* [50,58]. We used the 12 identified polymorphic EST-derived markers [45,50] and one SSR marker [46] to genotype all *P. halstedii* isolates. PCR amplification reactions were carried out as described by Ahmed et al. (2012) [46]. Briefly, a final volume of 25 µL containing 10 ng of genomic DNA, 2 mM of MgCl2, 150 µM of each dNTP, 4 pmol of each primer, and 0.2 U Taq Silverstar DNA polymerase (Eurogentec) was placed in a reaction buffer. Amplifications were performed with the following program: an initial denaturation step of 4 min at 96 °C, followed by 40 cycles of 40 s denaturation at 96 °C, 50 s annealing at 57 °C, 90 s elongation at 72 °C, and a final elongation step of 10 min at 72 °C.

Three SNPs, Pha54, Pha56, and Pha82, which had been transformed into cleaved amplified polymorphism sequence (CAPS) markers, were run as described by Giresse et al. (2007) [45]. The four SNPs, Pha6, Pha79, Pha99, and Pha120, were directly screened by PCR-SSCP, since no enzyme discriminating between the alleles could be found. Pha39, Pha42, Pha43, and Pha106 correspond to five insertion–deletion (indels) polymorphisms and the microsatellite (SSR-ph7) was run automatically on a Beckman Coulter Ceq8000 capillary sequencer. Pha74 was directly visualized on agarose gel.

The results of the molecular analysis were confirmed by using the positive control races (100, 703, and 710) that were previously typed and by repeating the analyses of 16 randomly selected isolates of *P. halstedii* using all the primer combinations. Repeated isolates were included in all the PCR runs in order to standardize allele scoring. The replicated profiles were compared, and markers with more than 5% error were removed from the datasets.

### 2.5. Data Analyses

Deviations from Hardy–Weinberg equilibrium for MLG (109) at the loci were tested with a chi-square test using the software GENEPOP 4.0 [59].

Genotyping the *P. halstedii* isolates with the 12 polymorphic EST-derived markers [45] and one SSR marker [46] allowed for their assignment to multilocus genotypes (MLG), sharing the same alleles at all loci. The calculations were performed for the overall dataset using the software GENCLONE 2.0 [60].

A fundamental prerequisite of any inference on the genetic structure of populations is the definition of the populations themselves. Because the genetic structure of populations is not always reflected in the geographical proximity of individuals, we investigated the genetic structure of *P. halstedii* isolates using the model-based Bayesian clustering approach of genetic mixture analysis (STRUCTURE 2.3.4 software). Simulations were performed using a dataset without multiple copies, from K = 1 to K = 6. For all the simulations, we did not force the model with predefined allele frequencies for source clusters. Five independent runs were conducted to assess the consistency of the results across runs, and all runs were based on 500,000 iterations after a burn-in period of 100,000 iterations. We followed the method developed by Evanno et al. (2005) [61] to identify the number of genetically homogeneous clusters (K).

The clonal diversity of each cluster was described, using GENCLONE, by the genetic richness (R) and the complement of the slope of the Pareto distribution of clonal membership, as recommended by Arnud-Haond and Belkhir (2007) [60], as the most parsimonious set of nonredundant indices of clonal diversity. In order to describe the intermingling of repeated MLG, we estimated the spatial aggregation index Ac as described by Arnud-Haond and Belkhir (2007) [60]. This index ranges from 0, when the probability between nearest neighbors does not differ on average from the global one, to 1, when all nearest neighbors preferentially share the same MLG. The statistical significance of the aggregation index was tested against the null hypothesis of spatially random distribution of isolates using a resampling approach based on 1000 permutations.

The inclusion of clonal multiple copies can strongly distort linkage disequilibrium between loci, genetic diversity, and other F-statistics. Standard population genetic tests were, therefore, performed without multiple copies using the software GENEPOP 4.0 [59]. Unbiased estimates of *F*_ST_ across loci were calculated according to Weir and Cockerham (1984) [62]. Allele frequencies and genetic diversity were calculated according to Nei et al. (1975) [63]. The number of pairs of loci showing significant linkage disequilibrium was determined by the exact test using the Markov chain algorithm of Raymond and Rousset (1995) [59]. In addition, we calculated the index of association (IA) to test for random recombination between pairs of all the loci for all *P. halstedii* isolates collected from this single field, as well as for the 109 multilocus genotypes. These tests were carried out using the software LIAN [64].

We performed principal coordinate (PCO) analysis using the molecular data to classify and detect the structure in the relationships between the isolates of different sites and clusters. These analyses were performed using R v2.9 [65].

Two separate molecular analysis of variance (AMOVA) tests [66] were performed, first for the partitioning of genetic variation among and within the 20 different sites, using all of the 250 *P. halstedii* isolates. The second was performed on 109 unique genotypes of *P. halstedii* to compare the two clusters. The mean *F*_ST_ was estimated in order to study the genetic differentiation between sites and the two clusters. The significance of *F*_ST_ values was tested by 1000 permutations. These analyses were performed using Arlequin software, version 2.000 [67]. We performed two Mantel tests [68]. First, Mantel’s test was used to correlate the matrix of genetic distance with the matrix of spatial distance. A geographic distance matrix was constructed using the distances in meters between the 20 sites in the field (GPS coordinates), while the genetic distance matrix was constructed using the genetic distance between each possible pair of the 250 isolates [69]. The geographic collection sites of the isolates based on their GPS coordinates were compared and correlated with the molecular analysis. A second Mantel’s test was carried out for the 64 isolates (the phenotypic data) of *P. halstedii* isolates (S = susceptible, R = resistant). We generated a quantitative data matrix as follows: R = 1 and S = 0. The data matrix was standardized and analyzed using the Manhattan and Euclid coefficients. Both analyses resulted in similar results, so only the results obtained with the Manhattan coefficient are presented. Then we compared and correlated these with the results from the molecular analysis of the corresponding *P. halstedii* isolates. The significance of Mantel’s test value was tested with 10,000 permutations; these analyses were performed using GENALEX 6.5 [70].

## 3. Results

The percentage of emerged seedlings of the GB sunflower line averaged 91% per site, with a range of 86–97% depending on the site. The percentage of infected seedlings was 34% on all 20 sites, ranging from 4% (site S16) to 54% (site S10). Only 4 pots out of 120 showed no diseased seedlings. This absence of symptoms is not related to an emergence problem, since 85–95% of the seedlings emerged in all pots (Table 2).

### 3.1. Phenotypic Diversity

Eight races were identified among the 64 *P. halstedii* isolates chosen for phenotyping collected from the same field (Table 3). The most frequently found races were 304 (70.3%) and 703 (15.6%), while the races present in low frequencies were race 100 (1.6%), 300 (1.6%), 307 (1.6%), 704 (3.0%), 707 (4.7%), and 714 (1.6%). The phenotypic analysis of *P. halstedii* isolates clearly showed that race 304 predominated, being present at 18 of the 20 sites (Table 3). However, the distribution of this race at the sites studied varied.

### 3.2. Genetic and Genotypic Diversity

Nine out of the thirteen molecular markers (SNPs and SSR) tested were polymorphic for the selected 250 *P. halstedii* isolates, while loci Pha42, Pha 54, Pha 56, and Pha 74 were monomorphic for all isolates (Table S1). The number of alleles detected with these markers in the 250 isolates analyzed was 36, of which 12 were detected using the single SSR pha-7. Most isolates from the field shared the most common alleles across the analyses. Based on the allele variation observed, 109 MLGs could be detected among the 250 *P. halstedii* isolates (Table 2). Sixty-three MLGs were represented by a single isolate, and 129 of the *P. halstedii* isolates belonged to five MLGs, i.e., MLGs 5, 91, 95, 96, and 99 (Table 3). The rest of the isolates belonged to 41 different MLGs, representing 2–5 isolates per MLG. The eight races detected in the 62 isolates of *P. halstedii* were split into 38 MLG, indicating high genotypic variability within the races. The 45 isolates of race 304 were split into 23 MLGs, while the 10 isolates of race 703 were split into 10 MLGs (results not shown). In general, the genotypic diversity found in this single small field was high. The analyses showed that isolates belonging to the same race had different genotypes, and isolates with identical alleles (same MLG) represented multiple races (Table 4).

**Figure 2:**
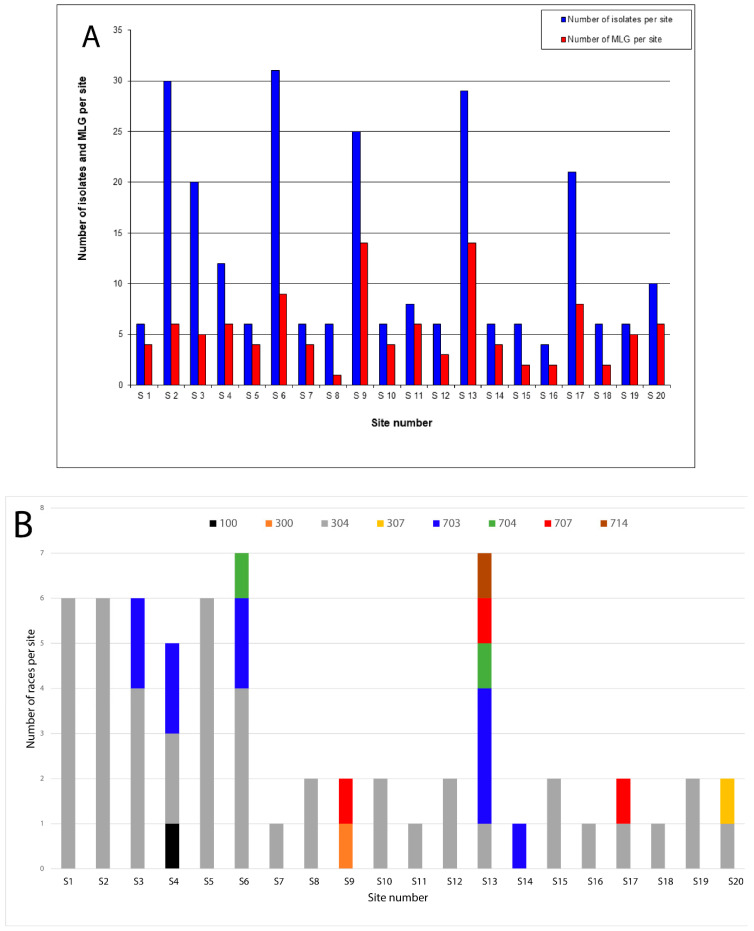
Distribution of *P. halstedii* within the field at the 20 sites under study. (**A**) Distribution of the total number of sunflower downy mildew isolates (250) and the number of MLG (109) at each site; (**B**) distribution of the eight races of *P. halstedii* (100, 300, 304, 307, 703, 704, 707, and 714) within the field at the 20 sites, with the co-existence of more than one race at one site (*n* = 64). (For interpretation of the references to color in this figure legend, the reader is referred to the web version of this article.).

It seems that an increased number of characterized isolates per site does not necessarily mean an increased number of MLG per sites. For example, at sites 2 and 3, we detected only 6 and 5 MLG out of 30 and 20 isolates, respectively, while at sites 11 and 19 we detected 6 and 5 MLG out of 8 and 6 characterized isolates, respectively (Table 3 and Figure 2).

The genotype richness was high (2.1600), and the genotypic diversity among the isolates was calculated based on the number of shared MLGs. Genetic diversity among the 20 sites characterized in this study varied from 0.0314 to 0.3298, with a mean of 0.1776 (Table 3). The *F*_ST_ value between the two clusters was very high at 0.3500.

The index of association among *P. halstedii* isolates (250) and MLG genotypes (109) was very low (IA = 0.1075, *p* < 0.0032 and IA = 0.0141, *p* < 0.0002 respectively), indicating random mating among *P. halstedii* in the field.

### 3.3. Principal Coordinate Analysis (PCO)

**Figure 3:**
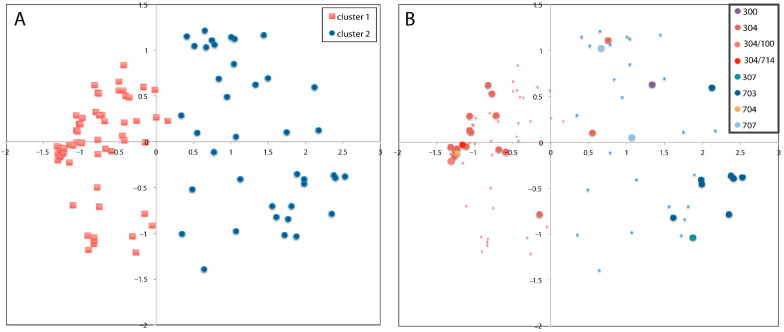
Plot of the principal coordinate analysis (PCO). (**A**) PCO of 109 unique genotypes shown on the first and second axis, with the proportion of ancestry from each cluster for each sample indicated by color: red shading is Cluster 1 and blue shading is Cluster 2; (**B**) distribution of the eight races of *P. halstedii* (100, 300, 304, 307, 703, 704, 707, and 714) within the field at the 20 sites (the figure shows the coexistence of more than one race at a site).

The PCO showed the distribution of the 250 *P. halstedii* within the field at the 20 sites in the study (Figure S1). PCO clustering of the isolates based on genetic similarity showed two main groups, which did not reflect the geographic sites of the isolates (Figure 3). Figure 3A is the PCO of 109 unique genotypes shown on the first and second axis, with the proportion of ancestry from each cluster for each sample indicated by color: blue shading is Cluster 1 and red shading is Cluster 2. Figure 3B shows the distribution of the eight races of *P. halstedii* (100, 300, 304, 307, 703, 704, 707, and 714) within the field at the 20 sites (the figure shows the coexistence of more than one race at one site) under study. The PCO revealed strong differentiation in the spread of the data, with 34.54% of the observed variation on the first axis and 18.69% on the second axis, with the data showing two distinct clusters. A single representative of each multilocus genotype was included in the PCO. This resulted in 109 different samples (Figure 3A). The eight races found in the study were grouped into two groups as follows: cluster 1 consisted of races 100, 304, 704, and 714, while cluster 2 comprised samples belonging to races 300, 307, 703, and 707 (Figure 3B and Table 4).

### 3.4. Individual Clustering Analysis

**Figure 4:**
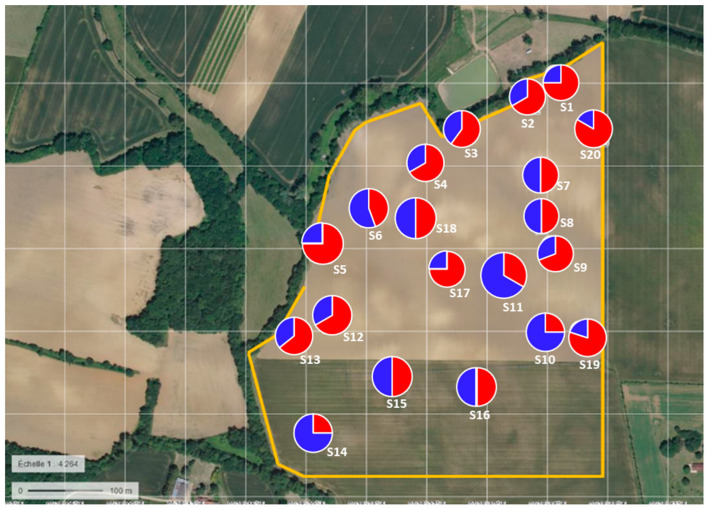
*P. halstedii* genotypes plotted in the field following their cluster assignment (C1 = red; C2 = blue) on the 20 sampling sites S1 to S20 (satellite image from https://www.geoportail.gouv.fr accessed on 5 September 2022).

Structural analysis simulated the maximum likelihood distribution L (K) at the real number of two groups (K = 2) (Figure 4). Importantly, this value was obtained using the value of ad hoc quantity (∆K) rather than maximum likelihood value L (K), as described by Evanno et al. (2005) [62]. The structural analysis clustered the *P. halstedii* isolates into two clusters (Figure S2).

Cluster 1 (C1) was made up of mostly 100, 304, 704, and 714. Cluster 2 (C2) consisted of races 300, 307, 703, and 707, as for the PCO groupings (Table 4). Summary statistics for the mean value across all loci for allelic richness and gene diversity were also calculated according to the two clusters assigned by structure (C1 and C2) (Table 4).

The results of the structural analysis supported the results of the PCO analysis. *P. halstedii* isolates were grouped into two main large clusters.

### 3.5. Clustering Differentiation

The global *F*_ST_ value for the two groups was high at 0.3500. The genetic diversity within each cluster was measured by the number of alleles per locus and the allelic richness (Table 5). Cluster 2 had 32 typed alleles in total and Cluster 1 had 26 alleles. A higher level of gene diversity, 0.1890, was observed in Cluster 2 (43 isolates, which are predominantly isolate 703) than in Cluster 1 at 0.0930 (66 isolates, predominantly isolate 304) (Table 5).

AMOVA analysis of *P. halstedii* between the two clusters (performed on 109 unique genotypes) showed high genetic variability among the clusters (65.11%) low genetic variability within the clusters (34.89%), and a high *F*_ST_ value (0.0.3500) (Table 6A). The global *F*_ST_ value for the two groups was high at 0.65 across all loci (*F*_IS_ 0.764), and observed patterns of differentiation were consistent across loci. AMOVA analysis between the 20 sites of the field (Table 6B) showed most of the total genetic variability within sites (95.34%), while the genetic variability between sites was very low (4.66%). Low genetic differentiation was detected among the 20 sites (*F*_ST_ = 0.0770).

### 3.6. Spatial Genetic Structure

The first Mantel’s test (geographical distances via molecular data) produced a very low value (r = 0.0015, *p* < 0.0017), and there was a lack of correlation between genetic data and geographic distances. The second Mantel test resulted in strong correlation and the genotypes plotted in the field following their cluster assignment, showing the coexistence of the two genetic clusters at this fine spatial scale (Figure 4). A significant correlation was detected between the molecular data and the phenotypic data (r = 0.6317, *p* < 0.0011).

## 4. Discussion

To our knowledge, this is the first study to describe the genetic diversity of *P. halstedii* in a single field by applying both phenotypic and molecular tools. In this study, we described the genetic characterization of 250 *P. halstedii* isolates collected from one field using molecular codominant markers. Molecular analysis of *P. halstedii* showed 109 genetic individuals among the 250 isolates collected in the field. Estimates of genetic relationships among *P. halstedii* isolates using a Bayesian clustering approach and PCO analysis divided the isolates into two main clusters. Analysis of molecular variance (AMOVA) revealed that most of the genetic variation was between clusters (65.11%), resulting in a high level of genetic differentiation (*F_ST_* = 0.3500). The two genetic clusters detected in the study may be due to two different introductions of *P. halstedii* populations in the area. The high levels of genetic differentiation between the genetic clusters indicated overall limited exchange between them, but our results also suggest that recombination between genotypes of these groups does occur.

The area where the isolates were collected was relatively small, but the results of this investigation provided definitive details about the population structure of *P. halstedii*. We detected a high level of genotypic diversity within the population of *P. halstedii* in this single field study compared to previous studies on *P. halstedii* [28,41,71]. If some of the genotypes found here have previously been detected in studies of French populations (reference strains collected each year), this study illustrates that the number of genotypes that can be detected at the field scale is much larger than expected. The frequency distribution of the different genotypes varied between the 20 sites investigated in the study. The low *F_ST_* value among the 20 sites indicated that isolates within the sites are genetically different, with each site containing multilocus genotypes that belong to the two clusters. The results, supported by the lack of correlations between genetic and geographic distances using Mantel’s test, indicate no spatial genetic structure of clusters at the scale of the field.

The second important result of the study is evidence for the co-existence of many different races of *P. halstedii* at the field level (phenotypic analyses indeed revealed the presence of eight different races in the field: 304, 714, 704, 100, 703, 307, 300, and 707). Among the eight races found in the field, the most abundant races were 304 (*n* = 45) and 703 (*n* = 10), corresponding to the races that are most frequently found in the south-west of France. It is worth noting that we did not detect races 710, 314, and 334 in this study because, in 2008, these races were restricted to northern regions in France. As previously demonstrated by Ahmed et al. (2012) [46], there was a strong association between races and the genetic structure observed in the field, as a result of several successive introductions of the pathogen in France. However, the interesting pattern found here is that these genetically differentiated races are coexisting at a very fine scale, allowing potential recombination of individuals. The large number of races detected in our study is in agreement with the regular discovery of new races in other countries in Europe and in North America [13,15,18,21,23,71,72,73,74].

This result is in accordance with previous studies that reported three genetic clusters using the same markers (Delmotte et al. (2008) and Ahmed et al. (2012) [46,50]): Cluster 1 contains races 100, 300, and 304; Cluster 2 contains races 307, 700, 703, 707 and 730; and Cluster 3 contains races 314, 334, 704, 710, 714, and 717. In this study, we only identified two of the genetic clusters previously described for this species by Delmotte et al. 2008 and Ahmed et al. 2012 [46,50], namely the groups centered on races 304 and 703. As mentioned above, race 710, which resulted from a third introduction of sunflower downy mildew in the north of France, was not present in this part of France in 2008. Our results are, therefore, in agreement with previous data that identified only two genetic clusters in the south of France (until the appearance of race 710 in this region) [46]. We can, therefore, deduce that races 704 and 714, belonging to Cluster 1 in our study, do not have the same origin as observed in previous studies, where these races clearly derived from race 710 (a race that is not present in the field). The same is true for race 300, which in our study was not genetically derived from race 100, as was the case in previous studies [46].

A high number of identical genotypes (141 out of 250; 56.4%) were detected in the field, indicating that clonal reproduction occurs in *P. halstedii* populations. Of course, we cannot exclude the possibility that these identical genotypes could also result from inbreeding, as *P. halstedii* is a homothallic species. Indeed, the high selfing rate observed in this study reduced genetic variation within the cluster and increased the linkage disequilibrium found in the three genetic groups with limited outcrossing. This may be a consequence of this species being homothallic with no known self incompatibility mechanism. Interestingly, we found evidence that at least three identical genotypes actually displayed different virulence profiles. The evolution of new races within a single clonal lineage has already been reported in oomycetes [46,75,76], and *P. halstedii* clonal lineages may have several different virulence profiles, as mentioned by Ahmed et al. (2012) [46] for races such as 100, 300, and 304 (Table S2).

Our results suggest that, although *P. halstedii* is mainly a homothallic species, recombination between different individuals is not rare, resulting in a large range of multilocus genotypes within and between genetic clusters. Using the relationship between *F*_IS_ and Selfing [*F*_IS_ = S/(2 − S)], we can estimate an outcrossing rate of about 6.5%. This is in line with our findings for the number of possible intermediate samples that may have arisen due to hybridization between different races (28 samples out of 109 unique genotypes), which makes the coexistence of races easier to envisage. In our study, a quarter of our samples were under the threshold of 70% of ancestry assigned to one or other cluster, and these samples were mainly races 304, 707, and 300. Another piece of evidence for outcrossing is that race 304, which was the most abundant in the field, is associated with several multilocus genotypes. This result confirms previous evidence that the same virulence profile can result from multiple recombination events. These results provide evidence that the recombination of individuals with different genetic backgrounds and virulence profiles does occur at a fine geographic scale. The hybridization scenario is also supported by the detection of races 304, 307, 704, and 714, which belong to a group of emergent races that had never been recorded outside France until 2014 [13]. Hybridization is also strongly suspected for race 707, which is thought to be a hybrid resulting from a cross between races 703 and 304 [51]. Novel pathogen genotypes arising from interspecific hybridization in natural populations are being documented more and more [46,77,78,79]. Milgroom et al. (2009) [80] and Spring and Zipper (2006; 2018) showed that recombination occurred between *Phytophthora* strains that were vegetatively incompatible. Finally, we must recall that recombination between races was already suspected by Tourvieille de Labrouhe et al. (2010) [81], who observed the development of new races (300, 304, 314, 700, 704, and 714) after infecting plots with only races 100 and 710. Although we do not have sufficient data to conclude that this mechanism has led to race emergence in situ, it appears to be a plausible explanation. Taken together, these results support the hypothesis that recombination between different individuals is an important evolutionary process in the *P. halstedii* population.

## Figures and Tables

**Table 1:** Races of *P. halstedii* as defined by the differential lines of sunflower (D1–D9).

Host	Virulence Profiles According to the Differential Sunflower Lines								
	D1	D2	D3	D4	D5	D6	D7	D8	D9
Score	1	2	4	1	2	4	1	2	4
races									
100	S ^1^	R ^2^	R	R	R	R	R	R	R
703	S	S	S	R	R	R	S	S	R
300	S	S	R	R	R	R	R	R	R
304	S	S	R	R	R	R	R	R	S
307	S	S	R	R	R	R	S	S	S
704	S	S	S	R	R	R	R	R	S
714	S	S	S	S	R	R	R	R	S
707	S	S	S	R	R	R	S	S	S

^1^ S: the host is susceptible (compatible interaction); ^2^ R: the host is resistant (incompatible interaction).

**Table 2:** Average percentages of emerged GB sunflower line seedlings and infected seedlings on the 20 sites.

Site	Percentage of Emerged Seedlings (%)	Percentage of Infected Seedlings (%)
1	95.0	33.0
2	92.5	41.6
3	93.3	22.1
4	93.3	12.0
5	90.8	31.8
6	92.5	43.1
7	91.7	35.5
8	90.8	45.9
9	85.8	49.1
10	92.5	54.1
11	90.8	18.7
12	92.5	38.3
13	87.5	39.1
14	89.2	30.7
15	89.2	16.7
16	95.0	4.3
17	91.7	26.5
18	96.7	48.0
19	90.0	22.1
20	89.2	15.5

**Table 3:** Genetic and phenotypic characteristics of *P. halstedii* samples for each site (S1–S20): total number of isolates, number of MLG, races, and genetic diversity.

Site	Number of Isolates	Number of MLG	Race	Genetic Diversity
S1	6	4	304_(6)_	0.1527
S2	30	6	304_(6)_	0.1908
S3	20	5	304_(4)_, 703_(2)_	0.1523
S4	12	6	100_(1)_, 304_(2)_, 703_(2)_	0.2635
S5	6	4	304_(6)_	0.0758
S6	31	9	304_(4)_, 703_(2)_, 704_(1)_	0.2045
S7	6	4	304_(1)_	0.3298
S8	6	1	304_(2)_	0.0314
S9	25	14	300_(1)_, 707_(1)_	0.2726
S10	6	4	304_(2)_	0.2913
S11	8	6	304_(1)_	0.2217
S12	6	3	304_(2)_	0.1433
S13	29	14	304_(1)_, 703_(3)_, 704_(1)_, 707_(1)_, 714_(1)_	0.2316
S14	6	4	703_(1)_	0.2540
S15	6	2	304_(2)_	0.0555
S16	4	2	304_(1)_	0.0576
S17	21	8	304_(1)_, 707_(1)_	0.1742
S18	6	2	304_(1)_	0.0629
S19	6	5	304_(2)_	0.1982
S20	10	6	304_(1)_, 307_(1)_	0.2465
Global	250	109	100_(1)_, 300_(1)_, 304_(45)_, 307_(1)_, 703_(10)_, 704_(2)_, 707_(3)_, 714_(1)_	0.1776

**Table 4:** Details of identical multilocus genotypes (MLG) among the genetic clusters. The five genotypes repeated more than five times are listed by cluster membership; the name of the MLG is denoted by a number, with the number of times it was observed in the full dataset, and in which races. Where the same genotype was found in more than one race, the number of samples for each race is given next to the race name in parenthesis.

	MLG Name	Number of Isolates	Race Characterization
Cluster 1	MLG91	26	304_(6)_, 714_(1)_
	MLG95	15	100_(1)_, 304_(3)_, 703_(1)_
	MLG96	48	304_(7)_, 704_(1)_
	MLG99	33	304_(8)_
Cluster 2	MLG5	7	703_(1)_

**Table 5:** Details and summary statistics for the genetic clusters defined by the program structure based on 13 nuclear loci. Cluster name, number of samples assigned to each cluster, number of unique genotypes, number of different races found in each cluster, names of races, mean allelic richness (Ar), gene diversity (Gd), and *F*_IS_ value (* *p* < 0.01).

Nuclear Cluster	Number of Isolates	Number of MLG	Number of Races	Races	Ar	Gd	*F*_IS_
Cluster 1	198	66	4	304, 714, 704, 100	1.54	0.093	0.815 *
Cluster 2	52	43	4	703, 307, 300, 707	2.05	0.189	0.682 *
Total	250	109	8		2.16	0.177	0.764 *

**Table 6:** (**A**). AMOVA analysis performed on 109 unique genotypes of *P. halstedii*. Comparisons are by cluster. (**B**). Analysis of molecular variance (AMOVA). AMOVA analysis of 250 *P. halstedii* isolates in the study. Comparisons are by site.

Regions	d.f.	Sum of Squares	Variance Components	% of Variation	*F*_ST_ Value
**A**					
Among clusters	1	254.331	3.25632	65.11	0.650
Within clusters	107	432.825	1.74526	34.89	
Total		687.156	5.00158		
**B**					
Among sites	19	81.792	0.13199	4.66	0.077
Within sites	230	621.684	2.70297	95.34	
Total		703.476	2.83496		

## Data Availability

Data supporting the reported results can be found at https://entrepot.recherche.data.gouv.fr/dataverse/inrae (accessed on 21 June 2022).

## Supplementary Materials

The following supporting information can be downloaded at: https://www.mdpi.com/article/10.3390/jof8101084/s1, Figure S1: Summary of the results of P. halstedii from the STRUCTURE analysis. The analysis was performed using the admixture model allowing for correlated frequencies: Electropherograms representing the two clusters found in the study (**A**). Dots plots representing the population assignment of the individuals for the assumption of delta K = 2 (**B**). Graph of LnP(K) (bars) for the different K popu-lation assumptions (**C**); Figure S2: Plot of the principal coordinate analysis (PCO) showing the distribution of the 250 *P. halstedii* within the field at the 20 sites in the study; Table S1: The 64 races, the Id number of the total of the 250 sunflower downy mildew isolates and the toltal numnber of alleles detected using the 13 molecular markers in the study; Table S2: Compare of the three clusters obtained by Delmotte et al. (2008) and Ahmed et al. (2012) with the two clusters detected in this study.
