# Supplementary material for: Spatial Genetic Structure and Pathogenic Race Composition at the Field Scale in the Sunflower Downy Mildew Pathogen, Plasmopara halstedii
Source: J Fungi (Basel). 2022 Oct 14;8(10):1084. doi: 10.3390/jof8101084 (PMC9605284; doi:10.3390/jof8101084)
Supplement: Supplementary file 1 [file jof-08-01084-s001.zip › jof-1806517-supplementary.pdf]

Article

Spatial genetic structure and pathogenic race composition at the field scale in the sunflower downy mildew pathogen, *Plasmopara halstedii*

Abdelhameed Elameen[1]*, Denis Tourvieille de Labrouhe[2], Emmanuelle Bret -Mestries[3], and Francois Delmotte[4]*

[1]NIOBIO, Norwegian Institute of Bioeconomy Research, Division of Biotechnology and Plant Health, N-1431 Ås, Norway

[2] Sainte-Anastasie, Le Bourg, 15170 Neussargues en Pinatelle, France

[3] Terres Inovia, Domaine de Magneraud, 17700 Saint-Pierre d'Amilly, France

[4] INRAE, Bordeaux Sciences Agro, ISVV, SAVE, 33140 Villenave d'Ornon, France

* Authors to whom correspondence should be addressed; e-Mail: abdelhameed.elameen@nibio.no and francois.delmotte@inrae.fr

Supplementary Table S1. The 64 races, the Id number *of the total of the 250 sunflower downy mildew isolates and the toltal numnber of alleles detected using the 13 molecular markers in the study.*

No	id	race	Pha6	Pha39	Pha42	Pha43	Pha54	Pha56	Pha74	Pha79	Pha82	Pha99	Pha106	Pha120	Ph7
1	h1217	304	200200	100100	100100	100100	100100	100100	100100	300300	100200	200200	200200	100100	205209
2	h1219	304	200200	200200	100100	100100	100100	100100	100100	300300	200200	200200	100100	200200	301301
3	h1221	304	200200	200200	100100	100100	100100	100100	100100	300300	200200	200200	100100	200200	297337
4	h1223	304	200200	200200	100100	100100	100100	100100	100100	300300	200200	200200	100100	200200	217217
5	h1225	304	200200	200200	100100	100100	100100	100100	100100	300300	200200	200200	100100	200200	297297
6	h1227	304	200200	200200	100100	100100	100100	100100	100100	300300	200200	200200	100100	200200	205307
7	h1229	304	200200	200200	100100	100100	100100	100100	100100	300300	200200	200200	100100	200200	311311
8	h1434		100100	100200	100100	100100	100100	100100	100100	300300	100200	100100	100200	100100	205307
9	h1435		100100	200200	100100	100100	100100	100100	100100	200200	200200	100100	200200	100100	205205
10	h1436		200200	200200	100100	100100	100100	100100	100100	300300	200200	200200	100100	200200	311311
11	h1437		200200	200200	100100	100100	100100	100100	100100	300300	200200	200200	100100	200200	301301
12	h1231	304	200200	200200	100100	100100	100100	100100	100100	300300	200200	200200	100100	200200	301301
13	h1439		200200	200200	100100	100100	100100	100100	100100	300300	200200	200200	100100	200200	311311
14	h1440		100100	100100	100100	100100	100100	100100	100100	200200	100200	100100	200200	100100	205205
15	h1441		200200	200200	100100	100100	100100	100100	100100	300300	200200	200200	100100	200200	321321
16	h1442		200200	200200	100100	100100	100100	100100	100100	300300	200200	200200	100100	200200	311311
17	h1233	304	200200	200200	100100	100100	100100	100100	100100	300300	200200	200200	100100	200200	217311
18	h1444		200200	200200	100100	100100	100100	100100	100100	300300	200200	200200	100100	200200	307307
19	h1445		200200	200200	100100	100100	100100	100100	100100	300300	200200	200200	100100	200200	301301
20	h1446		200200	200200	100100	100100	100100	100100	100100	300300	200200	200200	100100	200200	307307
21	h1447		100100	100100	100100	100100	100100	100100	100100	200200	100200	100100	200200	100100	205205
22	h1235	304	200200	200200	100100	100100	100100	100100	100100	300300	200200	200200	100100	200200	301301
23	h1449		200200	200200	100100	100100	100100	100100	100100	300300	200200	200200	100100	200200	301301

24	h1450		200200	200200	100100	100100	100100	100100	100100	300300	200200	200200	100100	200200	301301
25	h1451		100100	100100	100100	100100	100100	100100	100100	200200	100200	100100	200200	100100	205205
26	h1452		200200	200200	100100	100100	100100	100100	100100	300300	200200	200200	100100	200200	301301
27	h1237	304	200200	200200	100100	100100	100100	100100	100100	300300	200200	200200	100100	200200	307311
28	h1454		200200	200200	100100	100100	100100	100100	100100	300300	200200	200200	100100	200200	297297
29	h1455		200200	200200	100100	100100	100100	100100	100100	300300	200200	200200	100100	200200	311311
30	h1456		200200	200200	100100	100100	100100	100100	100100	300300	200200	200200	100100	200200	301301
31	h1457		200200	200200	100100	100100	100100	100100	100100	300300	200200	200200	100100	200200	311311
32	h1239	304	200200	200200	100100	100100	100100	100100	100100	300300	200200	200200	100100	200200	297297
33	h1459		200200	200200	100100	100100	100100	100100	100100	300300	200200	200200	100100	200200	301301
34	h1460		200200	200200	100100	100100	100100	100100	100100	300300	200200	200200	100100	200200	317317
35	h1461		200200	200200	100100	100100	100100	100100	100100	300300	200200	200200	100100	200200	297297
36	h1462		200200	200200	100100	100100	100100	100100	100100	300300	200200	200200	100100	200200	301301
37	h1241	304	200200	200200	100100	100100	100100	100100	100100	300300	200200	200200	100100	200200	311311
38	h1464		200200	200200	100100	100100	100100	100100	100100	300300	200200	200200	100100	200200	307307
39	h1465		200200	200200	100100	100100	100100	100100	100100	300300	200200	200200	100100	200200	307317
40	h1466		200200	200200	100100	100100	100100	100100	100100	300300	200200	200200	100100	200200	307307
41	h1467		200200	200200	100100	100100	100100	100100	100100	300300	200200	200200	100100	200200	307307
42	h1243	703	100100	100100	100100	100100	100100	100100	100100	200200	200200	100100	200200	100100	311311
43	h1469		200200	100100	100100	100100	100100	100100	100100	300300	200200	200200	100100	200200	205205
44	h1470		200200	200200	100100	100100	100100	100100	100100	300300	200200	200200	100100	200200	307307
45	h1471		200200	200200	100100	100100	100100	100100	100100	300300	200200	200200	100100	200200	297297
46	h1472		200200	200200	100100	100100	100100	100100	100100	300300	200200	200200	100100	200200	301301
47	h1245	304	200200	200200	100100	100100	100100	100100	100100	300300	200200	200200	100100	200200	297297
48	h1474		200200	200200	100100	100100	100100	100100	100100	300300	200200	200200	100100	200200	307307
49	h1247	703	100100	100100	100100	100100	100100	100100	100100	200200	100100	100100	200200	100100	205205
50	h1476		200200	200200	100100	100100	100100	100100	100100	300300	200200	200200	100100	200200	311311
51	h1477		200200	200200	100100	100100	100100	100100	100100	300300	200200	200200	100100	200200	307307
52	h1478		200200	200200	100100	100100	100100	100100	100100	300300	200200	200200	100100	200200	307311
53	h1249	304	200200	200200	100100	100100	100100	100100	100100	300300	200200	200200	100100	200200	205311
54	h1251	304	200200	200200	100100	100100	100100	100100	100100	300300	200200	200200	100100	200200	307307
55	h1481		200200	200200	100100	100100	100100	100100	100100	300300	200200	200200	100100	200200	307307
56	h1482		200200	200200	100100	100100	100100	100100	100100	300300	200200	200200	100100	200200	307307
57	h1253	304	200200	200200	100100	100100	100100	100100	100100	300300	200200	200200	100100	200200	307307
58	h1484		200200	100200	100100	100100	100100	100100	100100	300300	200200	200200	100200	200200	205205
59	h1255	304	200200	200200	100100	100100	100100	100100	100100	300300	200200	200200	100100	200200	307307
60	h1486		200200	200200	100100	100100	100100	100100	100100	200200	200200	100100	100200	100100	311311
61	h1257	703	100100	999	100100	999	100100	100100	100100	200200	100100	100100	200200	100100	205205
62	h1488		100100	200200	100100	100100	100100	100100	100100	300300	100200	200200	100100	200200	205311
63	h1489		200200	200200	100100	100100	100100	100100	100100	300300	200200	200200	100100	200200	307307
64	h1490		200200	200200	100100	100100	100100	100100	100100	300300	200200	200200	100100	200200	307307
65	h1491		200200	200200	100100	100100	100100	100100	100100	300300	200200	200200	100100	200200	307307

66	h1259	100	200200	200200	100100	100100	100100	100100	100100	300300	200200	200200	100100	200200	301301
67	h1493		100100	200200	100100	100100	100100	100100	100100	300300	200200	100100	100100	200200	307307
68	h1261	703	100100	100100	100100	100100	100100	100100	100100	200200	200200	100100	100100	100100	205205
69	h1263	304	200200	200200	100100	100100	100100	100100	100100	300300	200200	200200	100100	200200	999
70	h1265	304	200200	200200	100100	100100	100100	100100	100100	200200	200200	100100	100100	200200	297297
71	h1267	304	200200	200200	100100	100100	100100	100100	100100	300300	200200	200200	100100	200200	297297
72	h1269	304	200200	200200	100100	100100	100100	100100	100100	300300	200200	200200	100100	200200	307307
73	h1271	304	200200	200200	100100	100100	100100	100100	100100	300300	200200	200200	100100	200200	311311
74	h1273	304	200200	100100	100100	100100	100100	100100	100100	300300	200200	200200	100100	200200	297311
75	h1346	703?	100100	100100	100100	100100	100100	100100	100100	200200	100100	100100	200200	100100	205205
76	h1520		100100	100200	100100	100100	100100	100100	100100	200200	200200	100100	200200	100100	205307
77	h1521		200200	200200	100100	100100	100100	100100	100100	300300	200200	200200	100100	200200	307307
78	h1522		200200	200200	100100	100100	100100	100100	100100	300300	200200	200200	100100	200200	311311
79	h1523		200200	200200	100100	100100	100100	100100	100100	300300	200200	200200	100100	200200	311311
80	h1277	304	200200	200200	100100	100100	100100	100100	100100	300300	200200	200200	100100	200200	307307
81	h1525		200200	200200	100100	100100	100100	100100	100100	300300	200200	200200	100100	200200	307307
82	h1526		200200	200200	100100	100100	100100	100100	100100	300300	200200	200200	100100	200200	307307
83	h1527		100200	100200	100100	100100	100100	100100	100100	200200	100200	100200	100200	100100	205307
84	h1528		200200	100200	100100	100100	100100	100100	100100	300300	100200	200200	100100	200200	205307
85	h1362	703	100100	100100	100100	100100	100100	100100	100100	200200	100200	100100	200200	100100	205205
86	h1369	704	200200	200200	100100	100200	100100	100100	100100	300300	200200	200200	100100	200200	999
87	h1530		100100	100100	100100	100100	100100	100100	100100	300300	100200	100200	200200	200200	205311
88	h1531		200200	200200	100100	100100	100100	100100	100100	300300	200200	200200	100100	200200	307307
89	h1532		100100	100100	100100	100100	100100	100100	100100	300300	200200	200200	200200	200200	311311
90	h1533		200200	100100	100100	100100	100100	100100	100100	300300	200200	200200	200200	200200	205205
91	h1281	304	200200	200200	100100	100100	100100	100100	100100	300300	200200	200200	100100	200200	311311
92	h1535		200200	200200	100100	100100	100100	100100	100100	300300	200200	200200	100100	200200	311311
93	h1536		200200	200200	100100	100100	100100	100100	100100	300300	200200	200200	100100	200200	311311
94	h1537		200200	200200	100100	100100	100100	100100	100100	300300	200200	200200	100100	200200	311311
95	h1538		200200	200200	100100	100100	100100	100100	100100	300300	200200	200200	100100	200200	311311
96	h1283	304	200200	100200	100100	100100	100100	100100	100100	300300	200200	200200	100100	200200	307307
97	h1540		100200	200200	100100	100100	100100	100100	100100	300300	200200	100200	100200	200200	205307
98	h1541		200200	200200	100100	100100	100100	100100	100100	300300	200200	200200	100100	200200	297297
99	h1542		200200	200200	100100	100100	100100	100100	100100	300300	200200	200200	100100	200200	307307
100	h1543		100200	200200	100100	100100	100100	100100	100100	300300	200200	100200	100200	200200	205307
101	h1285	304	200200	200200	100100	100100	100100	100100	100100	300300	200200	200200	100100	200200	307307
102	h1545		200200	200200	100100	100100	100100	100100	100100	300300	200200	200200	100100	200200	307307
103	h1546		200200	200200	100100	100100	100100	100100	100100	300300	200200	200200	100100	200200	307307
104	h1547		200200	200200	100100	100100	100100	100100	100100	300300	200200	200200	100100	200200	297307
105	h1548		200200	200200	100100	100100	100100	100100	100100	300300	200200	200200	100100	200200	297297
106	h1287	304	200200	200200	100100	100100	100100	100100	100100	300300	200200	200200	100100	200200	311311
107	h1554		200200	200200	100100	100100	100100	100100	100100	300300	200200	200200	100100	200200	307307

108	h1559		100100	100100	100100	100100	100100	100100	100100	200200	100200	100100	200200	100100	205205
109	h1564		100100	100100	100100	100100	100100	100100	100100	200200	100200	100100	200200	100100	205205
110	h1566		100100	100100	100100	100100	100100	100100	100100	200200	100100	200200	200200	100100	205205
111	h1568		100100	200200	100100	100100	100100	100100	100100	300300	200200	100200	100200	200200	205307
112	h1289	304	200200	100200	100100	100100	100100	100100	100100	300300	200200	200200	100100	200200	307307
113	h1577		200200	200200	100100	100100	100100	100100	100100	300300	200200	200200	100100	200200	307307
114	h1291	304	200200	100100	100100	100100	100100	100100	100100	300300	200200	200200	100100	200200	307307
115	h1584		200200	200200	100100	100100	100100	100100	100100	300300	200200	200200	100100	200200	307307
116	h1589		200200	200200	100100	100100	100100	100100	100100	300300	200200	200200	100100	200200	307307
117	h1594		200200	200200	100100	100100	100100	100100	100100	300300	200200	200200	100100	200200	307307
118	h1361	300	100100	100100	100100	100100	100100	100100	100100	200200	200200	100200	200200	200200	205307
119	h1600		200200	200200	100100	100100	100100	100100	100100	300300	200200	200200	100100	200200	307307
120	h1601		200200	200200	100100	100100	100100	100100	100100	300300	200200	200200	100100	200200	297297
121	h1602		200200	200200	100100	100100	100100	100100	100100	300300	200200	200200	100100	200200	307307
122	h1354	707	200200	100100	100100	100100	100100	100100	100100	300300	100200	100100	200200	100100	311311
123	h1604		100100	100100	100100	100100	100100	100100	100100	200200	100200	100100	100100	100100	205205
124	h1605		100100	100100	100100	100100	100100	100100	100100	200200	100200	100100	200200	100100	205205
125	h1606		100200	100100	100100	100100	100100	100100	100100	200200	100200	100100	100200	100100	311311
126	h1607		200200	200200	100100	100100	100100	100100	100100	200200	200200	200200	100100	200200	297301
127	h1608		200200	200200	100100	100100	100100	100100	100100	200200	100200	100200	100200	200200	205307
128	h1609		100200	100200	100100	100100	100100	100100	100100	300300	200200	100200	100200	200200	205307
129	h1610		100100	200200	100100	100100	100100	100100	100100	300300	200200	200200	100100	200200	297297
130	h1611		100100	200200	100100	100100	100100	100100	100100	300300	200200	200200	100100	200200	307307
131	h1612		100100	100200	100100	100100	100100	100100	100100	300300	200200	100200	100200	200200	205307
132	h1613		200200	200200	100100	100100	100100	100100	100100	300300	200200	200200	100100	200200	297297
133	h1614		200200	200200	100100	100100	100100	100100	100100	300300	200200	200200	100100	200200	297297
134	h1615		100100	100100	100100	100100	100100	100100	100100	300300	200200	200200	200200	200200	205205
135	h1619		200200	200200	100100	100100	100100	100100	100100	300300	200200	200200	100100	200200	311311
136	h1620		200200	200200	100100	100100	100100	100100	100100	300300	200200	200200	100100	100100	307307
137	h1621		100100	100100	100100	100100	100100	100100	100100	300300	200200	100100	200200	200200	217217
138	h1622		100100	200200	100100	100100	100100	100100	100100	300300	200200	200200	100100	200200	307307
139	h1623		100100	200200	100100	100100	100100	100100	100100	300300	200200	200200	100100	100100	317317
140	h1624		200200	200200	100100	100100	100100	100100	100100	300300	200200	200200	100100	200200	217217
141	h1625		100100	200200	100100	100100	100100	100100	100100	300300	200200	200200	100100	100100	205205
142	h1626		200200	200200	100100	100100	100100	100100	100100	300300	200200	200200	100100	200200	307307
143	h1297	304	200200	200200	100100	100100	100100	100100	100100	300300	200200	100100	100100	200200	311311
144	h1299	304	200200	200200	100100	100100	100100	100100	100100	300300	200200	200200	100100	200200	311311
145	h1637		200200	200200	100100	100100	100100	100100	100100	300300	200200	200200	100100	200200	307307
146	h1642		100100	200200	100100	100100	100100	100100	100100	300300	200200	100100	100100	100100	311311
147	h1647		100100	100100	100100	100100	100100	100100	100100	200200	100200	100100	200200	100200	205205
148	h1651		100200	100100	100100	100100	100100	100100	100100	200200	200200	100100	200200	100200	205205
149	h1656		200200	100200	100100	100100	100100	100100	100100	200200	200200	100200	100200	100200	205311

150	h1301	304	200200	200200	100100	100100	100100	100100	100100	300300	200200	200200	100100	200200	307307
151	h1658		200200	200200	100100	100100	100100	100100	100100	300300	200200	100100	100100	100100	297297
152	h1662		200200	200200	100100	100100	100100	100100	100100	300300	200200	200200	100100	100100	305311
153	h1664		200200	200200	100100	100100	100100	100100	100100	300300	200200	200200	100100	100100	311311
154	h1669		200200	200200	100100	100100	100100	100100	100100	300300	200200	200200	100100	100200	297297
155	h1670		100100	100100	100100	100100	100100	100100	100100	300300	200200	100100	200200	100200	205205
156	h1671		200200	200200	100100	100100	100100	100100	100100	300300	200200	200200	100100	100200	205205
157	h1303	304	200200	100200	100100	100100	100100	100100	100100	300300	200200	200200	100100	200200	297297
158	h1305	304	200200	200200	100100	100100	100100	100100	100100	300300	200200	200200	100100	200200	311311
159	h1682		200200	200200	100100	100100	100100	100100	100100	300300	200200	200200	100100	200200	311311
160	h1687		200200	200200	100100	100100	100100	100100	100100	300300	200200	200200	100100	200200	297297
161	h1690		200200	200200	100100	100100	100100	100100	100100	300300	200200	200200	100100	200200	205205
162	h1692		100100	200200	100100	100100	100100	100100	100100	200200	200200	200200	200200	200200	305305
163	h1307	703	100100	100100	100100	100100	100100	100100	100100	200200	100100	100100	200200	200200	205205
164	h1695		100100	100200	100100	100100	100100	100100	100100	300300	200200	100100	200200	200200	205205
165	h1696		200200	200200	100100	100100	100100	100100	100100	300300	200200	200200	100100	200200	297297
166	h1697		200200	100100	100100	100100	100100	100100	100100	300300	200200	100100	200200	200200	311311
167	h1698		200200	100200	100100	100100	100100	100100	100100	300300	200200	100200	100200	200200	307311
168	h1309	707	200200	100100	100100	100100	100100	100100	100100	300300	100200	100100	200200	200200	311311
169	h1700		200200	200200	100100	100100	100100	100100	100100	300300	200200	200200	100100	100100	311311
170	h1701		200200	200200	100100	100100	100100	100100	100100	300300	200200	200200	100100	200200	311311
171	h1702		200200	100200	100100	100100	100100	100100	100100	300300	200200	100200	100200	200200	311311
172	h1703		100200	100200	100100	100100	100100	100100	100100	300300	200200	200200	100200	200200	307307
173	h1311	304	200200	200200	100100	100100	100100	100100	100100	300300	200200	100100	100100	200200	307307
174	h1705		100200	100200	100100	100100	100100	100100	100100	300300	200200	200200	100200	200200	307307
175	h1706		200200	200200	100100	100100	100100	100100	100100	300300	200200	200200	100100	200200	307307
176	h1707		100100	100200	100100	100100	100100	100100	100100	300300	100200	100100	100100	200200	217217
177	h1708		200200	100100	100100	100100	100100	100100	100100	300300	100200	100100	200200	200200	205311
178	h1313	703	200200	100100	100100	100100	100100	100100	100100	200200	100100	100100	200200	100100	205205
179	h1710		200200	100200	100100	100100	100100	100100	100100	300300	200200	200200	100200	200200	307307
180	h1711		200200	200200	100100	100100	100100	100100	100100	300300	200200	200200	100100	200200	311311
181	h1712		200200	200200	100100	100100	100100	100100	100100	300300	200200	200200	999	200200	307307
182	h1713		200200	200200	100100	100100	100100	100100	100100	300300	200200	200200	100100	200200	311311
183	h1343	703	100100	100100	100100	100100	100100	100100	100100	200200	100100	100100	200200	200200	205205
184	h1344	704	200200	200200	100100	100100	100100	100100	100100	300300	200200	200200	100100	200200	307307
185	h1715		200200	200200	100100	100100	100100	100100	100100	300300	200200	200200	100100	100100	307307
186	h1716		200200	200200	100100	100100	100100	100100	100100	300300	200200	200200	100100	200200	311311
187	h1717		200200	200200	100100	100100	100100	100100	100100	300300	200200	200200	100100	200200	307307
188	h1317	714	200200	200200	100100	100100	100100	100100	100100	300300	200200	200200	100100	200200	297297
189	h1719		200200	200200	100100	100100	100100	100100	100100	300300	200200	200200	100100	200200	307307
190	h1720		200200	200200	100100	100100	100100	100100	100100	300300	200200	200200	100100	200200	307307
191	h1721		100200	200200	100100	100100	100100	100100	100100	300300	200200	100200	100200	200200	307307

192	h1722		200200	200200	100100	100100	100100	100100	100100	300300	200200	200200	100200	200200	307307
193	h1319	703	100100	100100	100100	200200	100100	100100	100100	200200	100200	100100	200200	100100	205205
194	h1728		200200	200200	100100	100100	100100	100100	100100	300300	200200	200200	100100	200200	297297
195	h1733		200200	200200	100100	100100	100100	100100	100100	300300	200200	200200	100100	200200	307307
196	h1735		200200	200200	100100	100100	100100	100100	100100	300300	200200	200200	100100	100100	307307
197	h1740		200200	200200	100100	100100	100100	100100	100100	300300	200200	200200	100100	200200	301301
198	h1321	304	200200	200200	100100	100100	100100	100100	100100	300300	200200	200200	100100	200200	297297
199	h1750		999	200200	100100	100100	100100	100100	100100	300300	200200	200200	100100	200200	307307
200	h1323	304	200200	200200	100100	100100	100100	100100	100100	300300	200200	200200	100100	200200	311311
201	h1755		200200	200200	100100	100100	100100	100100	100100	300300	200200	200200	100100	200200	307307
202	h1757		200200	200200	100100	100100	100100	100100	100100	300300	200200	200200	100100	200200	311311
203	h1758		200200	200200	100100	100100	100100	100100	100100	300300	200200	200200	100100	200200	307307
204	h1760		200200	200200	100100	100100	100100	100100	100100	300300	200200	200200	100100	200200	307311
205	h1761		200200	200200	100100	100100	100100	100100	100100	300300	200200	200200	100100	200200	307307
206	h1325	304	200200	200200	100100	100100	100100	100100	100100	300300	200200	200200	100100	200200	297297
207	h1764		200200	200200	100100	100100	100100	100100	100100	300300	200200	200200	100100	200200	311311
208	h1327	304	200200	200200	100100	100100	100100	100100	100100	300300	200200	200200	100100	200200	317317
209	h1765		200200	200200	100100	100100	100100	100100	100100	300300	200200	200200	100100	200200	297297
210	h1767		200200	200200	100100	100100	100100	100100	100100	300300	200200	200200	100100	200200	297317
211	h1329	707	200200	200200	100100	100100	100100	100100	100100	300300	200200	200200	100100	200200	315315
212	h1769		200200	200200	100100	100100	100100	100100	100100	300300	200200	200200	100100	200200	311311
213	h1770		200200	200200	100100	100100	100100	100100	100100	300300	200200	200200	100100	200200	307311
214	h1771		100200	100200	100100	100100	100100	100100	100100	300300	200200	100200	100200	200200	205205
215	h1772		100200	100200	100100	100100	100100	100100	100100	300300	200200	200200	100100	200200	307307
216	h1773		200200	200200	100100	100100	100100	100100	100100	300300	200200	200200	100100	200200	297297
217	h1774		200200	200200	100100	100100	100100	100100	100100	300300	200200	200200	100100	200200	297297
218	h1775		200200	200200	100100	100100	100100	100100	100100	300300	200200	200200	100100	200200	297297
219	h1776		200200	200200	100100	100100	100100	100100	100100	300300	200200	200200	100100	200200	297297
220	h1777		200200	200200	100100	100100	100100	100100	100100	300300	200200	200200	100100	200200	301301
221	h1778		200200	200200	100100	100100	100100	100100	100100	300300	200200	200200	100100	200200	297297
222	h1779		100200	100200	100100	100100	100100	100100	100100	300300	200200	100200	100200	200200	205205
223	h1780		200200	100200	100100	100100	100100	100100	100100	300300	200200	200200	100100	200200	311311
224	h1781		100100	100100	100100	100100	100100	100100	100100	300300	100200	100100	100100	200200	205205
225	h1782		100100	100100	100100	100100	100100	100100	100100	300300	100200	100100	200200	200200	205205
226	h1783		100100	100200	100100	100100	100100	100100	100100	300300	200200	100100	200200	200200	205205
227	h1784		200200	200200	100100	100100	100100	100100	100100	300300	200200	200200	100100	200200	311311
228	h1785		200200	200200	100100	100100	100100	100100	100100	300300	200200	200200	100100	100100	305305
229	h1331	304	200200	100200	100100	100100	100100	100100	100100	300300	200200	200200	100100	200200	297297
230	h1791		200200	200200	100100	100100	100100	100100	100100	300300	200200	200200	100100	200200	297297
231	h1796		200200	200200	100100	100100	100100	100100	100100	300300	200200	200200	100100	200200	297297
232	h1801		200200	200200	100100	100100	100100	100100	100100	300300	200200	200200	100100	200200	311311
233	h1806		200200	200200	100100	100100	100100	100100	100100	300300	200200	200200	100100	200200	311311

234	h1811		200200	200200	100100	100100	100100	100100	100100	300300	100200	200200	100100	200200	297297
235	h1815		100100	200200	100100	100100	100100	100100	100100	300300	200200	100100	100200	200200	307307
236	h1819		200200	200200	100100	100100	100100	100100	100100	300300	200200	200200	100200	200200	205205
237	h1333	304	999	100200	100100	100200	100100	100100	100100	300300	200200	200200	100100	200200	311311
238	h1335	304	200200	100100	100100	100100	100100	100100	100100	300300	200200	100100	200200	200200	205205
239	h1828		200200	100100	100100	100100	100100	100100	100100	300300	200200	200200	100200	200200	311311
240	h1829		100100	200200	100100	100100	100100	100100	100100	300300	200200	200200	100100	200200	205205
241	h1832		200200	200200	100100	100100	100100	100100	100100	300300	200200	200200	100100	200200	307307
242	h1833		200200	200200	100100	100100	100100	100100	100100	300300	200200	200200	100100	200200	307307
243	h1337	304	200200	100200	100100	100200	100100	100100	100100	300300	200200	200200	100100	200200	297297
244	h1835		200200	200200	100100	100100	100100	100100	100100	300300	200200	200200	100100	200200	307307
245	h1836		100100	200200	100100	100100	100100	100100	100100	300300	100200	100100	200200	200200	205205
246	h1837		200200	200200	100100	100100	100100	100100	100100	200200	200200	100100	200200	200200	205205
247	h1838		100200	100200	100100	100100	100100	100100	100100	300300	200200	200200	100200	200200	205205
248	h1339	307	100100	200200	100100	100100	100100	100100	100100	200200	100200	100100	200200	100100	205205
249	h1840		200200	200200	100100	100100	100100	100100	100100	300300	200200	200200	100100	200200	311311
250	h1841		200200	200200	100100	100100	100100	100100	100100	300300	200200	200200	100100	100100	297297

Supplementary Table S2. Compare of the three clusters obtained by Delmotte et al. (2008) and Ahmed et al. (2012) with the two clusters detected in this study.

Delmotte et al. (2008) and Ahmed et al. (2012)			This study (with only the first two cluster)	
cluster 1	cluster 2	cluster 3	cluster 1	cluster 2
100	307	314	100	300*
300	700	334	**304**	307
**304**	**703**	704	704*	**703**
	707	**710**	714*	707
	730	714		

* in this study, races 704, 714 and 300 have a different origin that those found in the previous studies (see discussion).

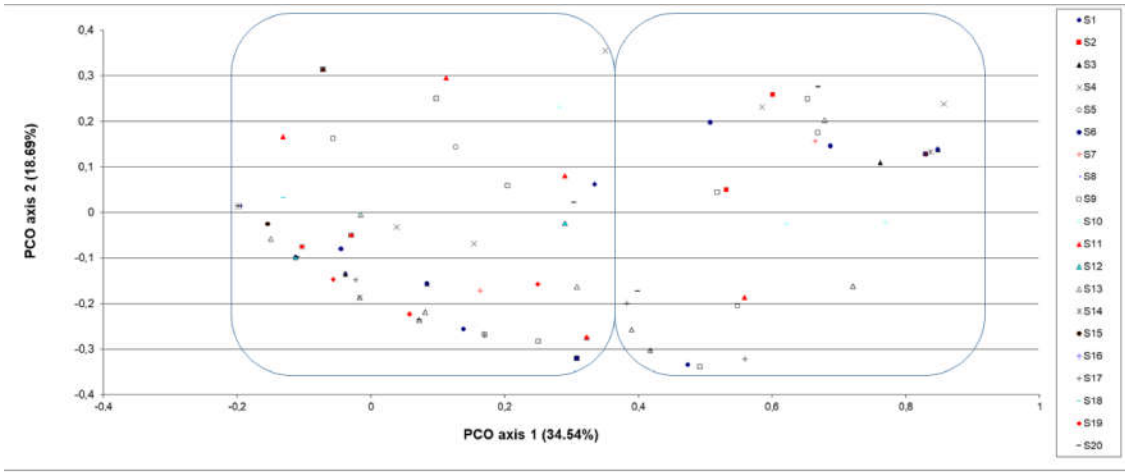

Supplementary Figure S1. Plot of the principal coordinate analysis (PCO) showing the distribution of the 250 *P. halstedii* within the field at the 20 sites in the study.

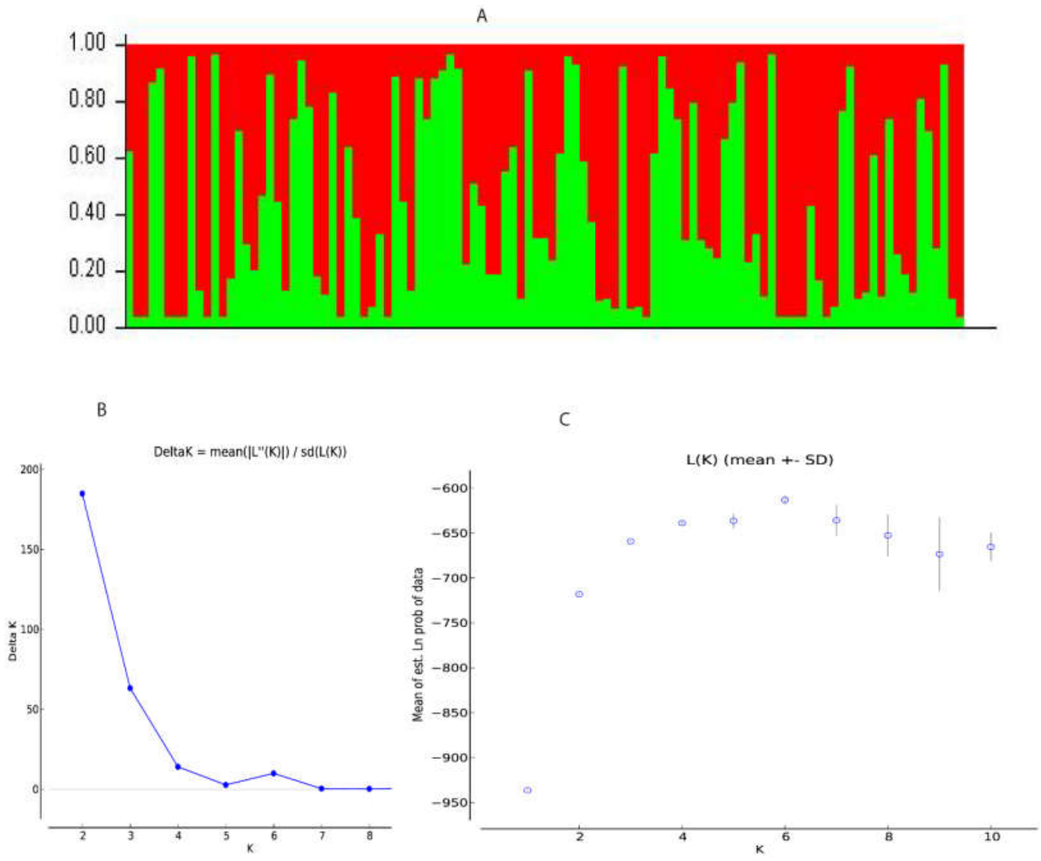

Supplementary Figure S2. Summary of the results of *P. halstedii* from the STRUCTURE analysis. The analysis was performed using the admixture model allowing for correlated frequencies: Electropherograms representing the two clusters found in the study (A). Dots plots representing the population assignment of the individuals for the assumption of delta K = 2 (B). Graph of LnP(K) (bars) for the different K population assumptions (C).
